# First order, then wash your hands: quantification and identification of bacteria on self-ordering touchscreens in fast food restaurants at times of high and low occupancy

**DOI:** 10.1186/s13104-026-07704-x

**Published:** 2026-02-09

**Authors:** Nadja Schwendenmann, Selina Fritz, Markus Egert

**Affiliations:** Faculty of Health, Medical and Life Sciences, Institute of Precision Medicine, Microbiology and Hygiene Group, Furtwangen University, Villingen-Schwenningen, Germany

**Keywords:** Touchscreen, Fast food, *Staphylococcus*, Hygiene

## Abstract

**Objective:**

Frequently touched public surfaces represent fomites. Interestingly, little is known about the microbial contamination of self-ordering touchscreens in fast food restaurants, despite the fact that food is usually consumed with hands shortly after ordering. To bridge this gap, we investigated the bacterial load of self-ordering touchscreens by surface sampling and subsequent identification of dominant morphotypes using MALDI-biotyping. We repeatedly (*n* = 5) sampled touchscreens in three different restaurants in the greater area of Villingen-Schwenningen, Germany, at times of low (≤ 5 guests) and high occupancy (≥ 20 guests), respectively.

**Results:**

Sampling with Violet Red Bile Agar plates indicated no contamination with Enterobacteriaceae. Sampling with Tryptic Soy Agar plates showed contamination for 28 out of 30 investigated screens, ranging from about 0.07 to 5.7 CFU/cm^2^ (absolute minimum and maximum, respectively). For all restaurants, median bacterial contamination during high occupancy times was 2.8 to 7.5 times higher than during low occupancy. Across all samples, 126 isolates were identified at least on genus level. Samples were dominated by staphylococci and micrococci, indicating human skin microbiota as main source of contamination. The results underline the need for regular touchscreen cleaning and hand hygiene before the meal, in particular at times of high occupancy.

## Introduction

Surfaces that regularly get in contact with humans and the human skin are usually contaminated with a diverse microbial community. Hence, such surfaces represent fomites and can contribute to the transmission of infectious disease [1]. This is of particular relevance in clinical environments [2]. In domestic and public environments, surfaces accessed by multiple individuals are generally of greater hygienic concern than private surfaces that are touched by only a single person.

For instance, previous investigations have demonstrated that commonly shared objects, such as door handles, elevator buttons, automated teller machines (ATMs), and money are significantly contaminated with bacteria, including potentially pathogenic species [3–8].

A survey of microbial contamination on restaurant non-food contact surfaces reported highest contaminations of up to 184 CFU/100 cm^2^ of total microbes detected on table chairs and 59 CFU/100 cm^2^ of Enterobacteriaceae on cleaning dishcloths [9]. A study assessing microbial contamination of self-service devices in Saudi Arabia found 78% of all investigated items to be contaminated, with *Escherichia coli* as the most prevalent bacterial contaminant [10]. In recent years, the use of self-ordering touchscreens has become increasingly popular in the fast food industry [11]. Despite their frequent use by numerous individuals throughout the day, little is known about the microbial burden of these surfaces. A PubMed search conducted on 04-10-25 using the keywords “fast food”, “touchscreen”, and “bacteria” yielded no results.

The hygienic status of these touchscreen surfaces is of particular interest, as fast food is usually consumed with bare hands shortly after ordering. In order to bridge this knowledge gap, we conducted a pilot study in which we repeatedly assessed the microbial load of self-ordering touchscreens using RODAC (Replicate Organism Detection and Counting) surface sampling. Sampling was performed in three different fast food restaurants in the greater area of Villingen-Schwenningen (Germany) at times of varying customer occupancy. Representative microbial isolates were subsequently identified by MALDI biotyping. The findings of this study may contribute to improved hygiene management of these increasingly popular ordering interfaces.

## Materials and methods

Surface samples were taken repeatedly (*n* = 5 sampling dates) over the course of about 5 weeks in three different fast food restaurants (R 1–3; belonging to three different operators, respectively) in the greater area of Villingen-Schwenningen (Germany), i.e., within a radius of 50 km around the city, in the summer of 2025. Due to the exploratory nature of this study, the relatively small sample size was considered sufficient. At each sampling date, the same self-ordering touchscreen was sampled using one RODAC plate with Tryptic Soy Agar (TSA) medium and one with Violet Red Bile Agar with glucose (VRBG) medium (both Carl Roth, Karlsruhe, Germany). According to the manufacturer, the plates had a diameter of 65 mm and a corresponding contact area of 28 cm^2^. The plates were firmly pressed on the surface of the centre of the screen for ca. 10 s, immediately brought to the laboratory, and incubated at 37 °C for 2–3 days until no more new colonies occurred. At each sampling date, one sampling was performed during a period of low occupancy, defined as a period with ≤ 5 guests in the restaurant, and another one during a period of high occupancy, defined as ≥ 20 guests in the room, respectively. All samplings were performed by the same person. Unused plates served as negative controls and were incubated as well, but did not show any microbial growth.

After incubation, colonies on each RODAC plate (30 TSA plates, 30 VRBG plates) were counted and germ numbers expressed as CFU/cm^2^. From each plate, a representative of each colony morphotype was used for identification using a Bruker MALDI Biotyper IVD System (Bruker Daltonics, Bremen, Germany) following the manufacturer’s instructions. In brief, colonies were carefully picked directly from the RODAC plates with sterile toothpicks, smeared onto the respective spots of a 96-spot Biotyper steel target, overlayed with 1 µl of α-cyano-4-hydroxycinnamic acid matrix (Bruker), dried for 2 min and then analysed. The obtained spectra were analysed using the software flexControl 3.4, MBT Compass (Version 4.1.100), and the MALDI Biotyper Compass database for bacteria and filamentous fungi (Version 4.1.100) (all Bruker). Following the instructions of the manufacturer and several other publications, identification scores ≥ 1.7 were counted as secure identification on genus level and scores ≥ 2.0 as secure identification on species level [12–14].

Germ count data were visualized as box-and-whisker plots with R (version 4.5.1) using the packages ‘dplyr’ and ‘ggplot2’ [15, 16]. Statistical comparisons between microbial loads were performed using R and non-parametric tests. Comparisons between the three different restaurants at times of high and low occupancy, respectively, were performed with H-tests (Kruskal-Wallis-tests), pair-wise comparisons between the data for low and high occupancy for each restaurant were done with U-tests (Wilcoxon-Mann-Whitney-tests). The p-values were corrected for multiple comparisons with the Bonferroni correction.

## Results

Germ counts obtained with VRBG contact agar plates indicated no contamination with Enterobacteriaceae across all samples. Just two colonies were detected on 30 used plates and identified as *Paracoccus yeei* and *Pseudomonas fulva*, respectively.

**Fig. 1:**
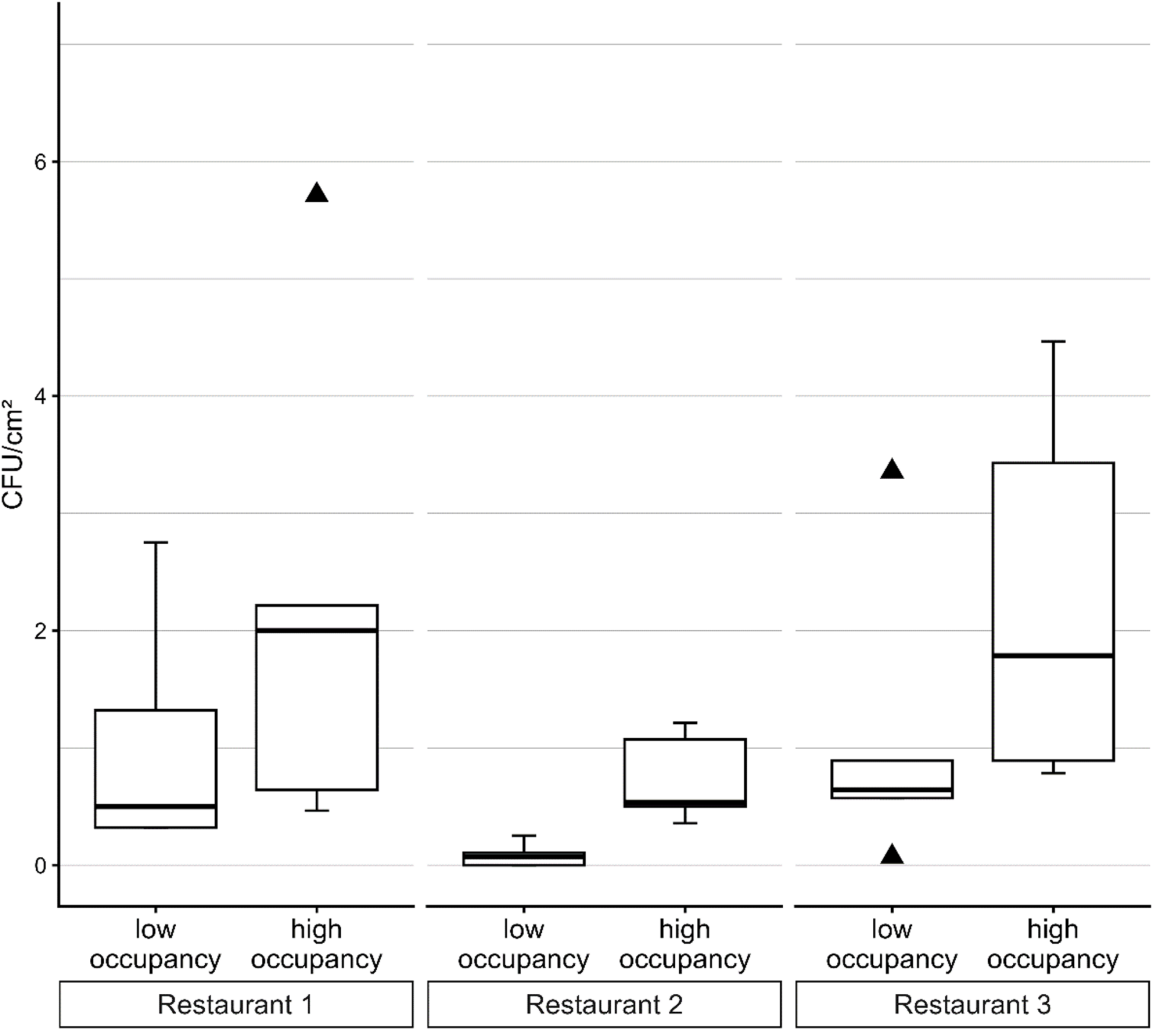
Box-and-whisker plot of the observed colony forming units per square centimeter (CFU/cm^2^) on TSA agar. Ordering terminals of three different fast food restaurants (Restaurant 1 to 3) were sampled when ≤ 5 customers were present (“low occupancy”; *n* = 5, each) and when ≥ 20 customers were present (“high occupancy”; *n* = 5, each). The horizontal bar within each box denotes the median. The upper and lower hinges correspond to the first and third quartile, respectively. The upper and lower whiskers extend from the hinges no further than 1.5x inter-quartile range (distance between first and third quartile). Outliers are data points beyond the range of the whiskers and shown as black triangles

In contrast, 28 out of 30 TSA contact agar plates showed microbial growth. Figure 1 displays the observed germ counts across the three restaurants during different times of occupancy as a box-and-whisker plot.

Overall, germ counts on TSA medium ranged from about 0.07 to 5.7 CFU/cm^2^ (absolute minimum and maximum, respectively). Median germ counts for low/high occupancy were 0.5/2.0 (R 1), 0.07/0.54 (R 2), and 0.64/1.79 (R 3) CFU/cm^2^, respectively. Overall, germ counts measured at levels of high and low occupancy were significantly different (*p* = 0.0105). However, pairwise comparisons revealed no significant differences for R 1 (*p* = 0.8844) and R 3 (*p* = 0.3482), but for R 2 (*p* = 0.0358). Median germ counts at times of high occupancy were 2.8 (R 3), 4.0 (R 1), and 7.5 (R 2) times higher than during times of low occupancy, respectively.

From each TSA plate with microbial growth (*n* = 28), one colony per morphotype was identified by means of MALDI biotyping. In total, 124 TSA isolates were analysed. 78 (62.9%) were securely analysed on species level, and 46 (37.1%) were at least securely analysed on genus level. Table 1 provides an overview about the identified taxa.

**Table 1:** Bacterial taxa isolated from TSA plates and identified with a MALDI biotyper score of > 1.7 (genus level) or > 2.0 (species level)

Phylum	Genus	Species	Number	Risk group
*Bacillota*	*Staphylococcus*	*epidermidis*	17	2
		*capitis*	14	1
		*hominis*	7	2
		*aureus*	6	2
		*haemolyticus*	1	2
		*lugdunensis*	1	2
		*saprophyticus*	1	2
		sp.	29	n. a.
	*Bacillus*	*cereus* group	1	n. a.
		sp.	2	n. a.
	*Priestia*	*megaterium*	1	1
	*Streptococcus*	sp.	1	n. a.
*Actinomycetota*	*Micrococcus*	*luteus*	21	1
		sp.	8	n. a.
	*Kocuria*	sp.	4	n. a.
	*Brevibacterium*	*casei*	2	2
		*celere*	1	1
	*Corynebacterium*	sp.	1	n. a.
*Pseudomonadota*	*Moraxella*	*osloensis*	3	2
		sp.	1	n. a.
	*Neisseria*	*sicca* group	1	2
	*Pseudomonas*	*massiliensis*	1	n. a.

Risk groups are based on the German TRBA 466 (Technical rules for biological agents 466 [17])

n. a.: not applicable

The three most frequently detected species were *Micrococcus luteus*, *Staphylococcus epidermidis*, and *Staphylococcus capitis*. Classification into risk groups was based on the German TRBA 466 (Technical Rules for Biological Agents 466 [17]). From 78 isolates securely identified on species level, 39 (50%) were classified as belonging to risk group 2, i.e., as potential pathogens.

## Discussion

To the best of our knowledge, this small-scale pilot study represents the first published study on the microbial contamination of self-ordering touchscreens in fast food restaurants. Our data show that these screens are largely contaminated with bacteria of human skin origin.

The observed median germ counts of 0.07 to 2.0 CFU/cm^2^ are similar to the microbial load of other non-food contact surfaces previously determined for restaurants and other frequently touched screens, such as smartphone touchscreens [9, 18, 19]. Interestingly, the germ counts appear lower than counts previously reported for conventional (paper) restaurant menus, for which up to 100 CFU/cm^2^ of aerobic bacteria have been reported [20]. We hypothesize that the very smooth surface of touchscreens and more regular cleaning measures might be responsible for this. Another study reported germ counts similar to ours for restaurant menus and a significant increase of this contamination when times of low and high occupancy were compared, also similar to what we have reported here [21].

Based on the identified species – predominantly staphylococci and micrococci – it is safe to assume that human hands and skin are the most important source of contamination of self-ordering touchscreens. A very similar bacterial community was reported for smartphone touchscreens using RODAC sampling and MALDI biotyping for the identification of dominant morphotypes [18].

Notably, no Enterobacteriaceae were detected on the investigated touchscreens, which is a positive result from a hygienic point of view and contradicting some alarmist older online reports [22]. Just two bacterial colonies were detected on the used VRBG plates (*n* = 30), however, they were securely not representing Enterobacteriaceae or faecal bacteria, but environmental bacteria. It is well known that occasionally non-enteric bacteria can (poorly) grow on VRBG plates [23]. Admittedly, as Enterobacteriaceae are often detected on human hands and therefore also frequently touched surfaces, it is safe to assume that Enterobacteriaceae might also occur on self-ordering touchscreens, presumably in restaurants with higher occupancies than the ones investigated here [5, 24].

Routine cleaning and/or disinfection measures were not observed and/or investigated in this study. However, our results suggest that they are sufficient to keep the level of contamination similar to other touchscreen and restaurant surfaces. Nonetheless, potentially pathogenic (risk group 2) species, such as *S. aureus*, were occasionally identified.

From a customer point of view, hand washing after ordering and before eating seems advisable to prevent microbial infections stemming from the touchscreens, in particular during peak times. In addition, cleaning the touchscreens prior to ordering with a wet lens wipe cloth can help reducing the microbial load by 1–2 log scales, which has been demonstrated for smartphone touchscreens and microscope ocular surfaces [18, 25].

## Conclusions

Our small-scale pilot study shows that self-ordering touchscreens in fast food restaurants are largely contaminated with bacteria of human skin origin, in particular during times of high occupancy. Consequently, these screens need to be cleaned and disinfected regularly by the restaurant staff. In addition, customers should be aware of the contamination, which is of particular concern as fast food is usually consumed with bare hands shortly after ordering. Pre-ordering disinfection with a wet lens wipe or similar products as well as hand hygiene before eating appear advisable.

## Limitations

Our study has limitations with respect to the small number of analysed samples, which also impacts statistical power. Clearly, follow-up studies should include more restaurants and more sampling dates. In addition, the distribution of bacteria across a self-ordering touchscreen might be different, hence, sampling one screen at different spots might be advisable. The contamination in highly frequented restaurants, e.g., in major cities, might be different compared to the restaurants in the greater area of Villingen-Schwenningen investigated here. Finally, our study was focused on the detection of aerobic, mesophilic bacteria associated with humans. Clearly, other cultivation and incubation conditions or the use of cultivation-independent, molecular techniques might be helpful to detect additional taxa on the screens, such as environmental bacteria, low abundant pathogens or highly contagious viruses, such as norovirus.

## Data Availability

All data are available from the corresponding author upon reasonable request.

## Abbreviations

ATM: Automated teller machine
CFU: Colony forming units
MALDI: Matrix-assisted laser desorption/ionization
R 1: Restaurant 1
R 2: Restaurant 2
R 3: Restaurant 3
RODAC: Replicate Organism Detection and Counting
TRBA: Technical Rules for Biological Agents
TSA: Tryptic Soy Agar
VRBG: Violet Red Bile Agar with glucose
